# Harnessing Natural Killer Cell Innate and Adaptive Traits in HIV Infection

**DOI:** 10.3389/fcimb.2020.00395

**Published:** 2020-08-04

**Authors:** Aljawharah Alrubayyi, Ane Ogbe, Elia Moreno Cubero, Dimitra Peppa

**Affiliations:** ^1^Nuffield Department of Clinical Medicine, University of Oxford, Oxford, United Kingdom; ^2^Peter Medawar Building for Pathogen Research, Nuffield Department of Medicine, University of Oxford, Oxford, United Kingdom; ^3^Department of HIV, Mortimer Market Centre, CNWL NHS Trust, London, OH, United Kingdom

**Keywords:** natural killer (NK) cells, human immunodeficiency virus (HIV), cytomegalovirus (CMV), adaptive NK cells, immunotherapy

## Abstract

Despite efficient virological suppression on antiretroviral therapy (ART), people living with HIV (PLWH), experience an increased burden of premature co-morbidities, such as cancer and end-organ disease. With remaining challenges in terms of access to therapy, adherence and potential long-term drug toxicity, improving their long-term healthcare outcome, including new strategies for HIV clearance, remains a global priority. There is, therefore, an ongoing need to better characterize and harness the immune response in order to develop new strategies and supplement current therapeutic approaches for a “functional” cure. Current efforts toward HIV eradication to enhance immune recognition and elimination of persistently infected cells have highlighted the need for an optimized “kill” approach. Natural killer (NK) cells play an important role in antiviral defense and by virtue of their innate and adaptive features hold great promise as a focus of “kill” efforts. Galvanized by advances in the cancer field, NK cell exploitation, represents a transformative approach to augment HIV therapeutic modalities, circumventing many of the limitations inherent to T cell approaches. In this review we will discuss recent advances in our understanding of the development of NK cell adaptive/memory responses in HIV infection and highlight new and exciting opportunities to exploit the beneficial attributes of NK cells for HIV immunotherapy.

## Overview of the Function of nk Cells and Role in HIV Infection

NK cells are multipotent innate effector cells that play pivotal roles in antiviral and tumor immunity (Vivier et al., 2008). They can rapidly eliminate virus-infected or transformed cells through contact dependent mechanisms and exocytosis of cytotoxic granules and/or via death receptor pathways that induce apoptosis (Vivier et al., 2008). Another important mechanism for the elimination of target cells by NK cells is antibody-dependent cell cytotoxicity (ADCC), mediated by the FcgRIIIA receptor (CD16), which binds the constant region (Fc) of immunoglobulin-opsonized cells. This interaction induces phosphorylation of the immunoreceptor tyrosine-based activation motif (ITAM) domains of the high-affinity IgE receptor (FcεRIγ) and CD3ζ in NK cells, and initiates a signaling cascade that ultimately results in the killing of the antibody-coated cell (Bournazos et al., 2017). In addition to cytotoxic elimination of target cells, NK cells are potent producers of cytokines and chemokines with antiviral function (Lodoen and Lanier, 2006). It is increasingly recognized that NK cells have an important immunoregulatory role with the ability to promote or suppress adaptive (i.e., T and B cells) and innate immune cells (i.e., dendritic cells) and influence the outcome of infection (Walzer et al., 2005; Waggoner et al., 2016). We have previously demonstrated the rheostat role of NK cells in human chronic viral infections, with the capacity to restrain antiviral immunity in chronic HBV infection (Peppa et al., 2013), and more recently the ability to regulate the development of broadly neutralizing antibodies (bNAbs) in HIV infection (Bradley et al., 2018).

NK cell activation is tightly regulated by the integration of signals from an array of germline-encoded inhibitory and activating receptors (Long et al., 2013). This dynamic balance ensures self-tolerance, whilst permitting robust responses against virally infected cells that have downregulated major histocompatibility complex class I (MHC I) molecules that ligate NK-expressed inhibitory receptors, such as killer immunoglobulin-like receptors (KIRs) and CD94/NKG2A, and/or upregulated stress ligands or viral associated molecules recognized by activating receptors, such as NKG2D or natural cytotoxicity receptors (NCRs) (Lanier, 2008; Orr and Lanier, 2010). During NK cell development, the interaction between inhibitory receptors and self-MHC molecules is critical for promoting their education and fine tuning their level of responsiveness (Elliott and Yokoyama, 2011; Boudreau and Hsu, 2018).

NK cells precede adaptive immunity during the early stages of HIV infection, where a rapid expansion of cytotoxic CD56^dim^ NK cells is observed prior to CD8 T cell expansion (Alter et al., 2007a). Evidence from immunogenetic, antiviral functional, and viral evolution/immune evasion studies further implicate NK cells as important contributors to immune control of HIV, linking specific KIR/HLA combinations with disease outcome and protective KIRs with enhanced NK cell function *in vitro* (Martin et al., 2002, 2007; Alter et al., 2007b; Shah et al., 2010). Further studies have indicated that the relative contribution of NK cells to control of viral replication is influenced by the degree of HIV-mediated changes to MHC class I expression and the strength of KIR/HLA interactions (Boudreau et al., 2016; Korner et al., 2017). Moreover, indirect NK cell-mediated ADCC is a potent means of control of HIV infection and has been associated with vaccine induced protective immunity and implicated in phenotypes of viral control and slower disease progression (Haynes et al., 2012; Wren et al., 2013; Kulkarni et al., 2017; Madhavi et al., 2017).

Whereas chronic HIV infection is well-documented to affect NK cell subset redistribution and functional ability (Mavilio et al., 2003; Fauci et al., 2005; Brunetta et al., 2010), these defects appear to be at least partially recovered following introduction of effective ART (Frias et al., 2015; Mikulak et al., 2017). More recently in treated HIV infection, phenotypic alterations in peripheral NK cells were not found to result in improved functional responses to HIV (Zhao et al., 2020). Thus, in ART-treated PLWH, targeting NK cell subsets to boost their range of antiviral properties and/or recover any residual dysfunction could improve control of HIV and restraint the development of detrimental co-morbidities.

With recent advances increasing our understanding of the anatomic control of NK cell development (Dogra et al., 2020) including potential for memory responses (O'Sullivan et al., 2015), the opportunities to direct and exploit these distinct features of NK cells to target HIV have grown. Here, we will consider current immunotherapeutic approaches to harness NK cells, highlighting the beneficial attributes of adaptive/memory NK cell subsets and potential advantage over their conventional counterparts.

## NK Cell-Based Strategies for Elimination of HIV—Learning From the Cancer Field

**Figure 1:**
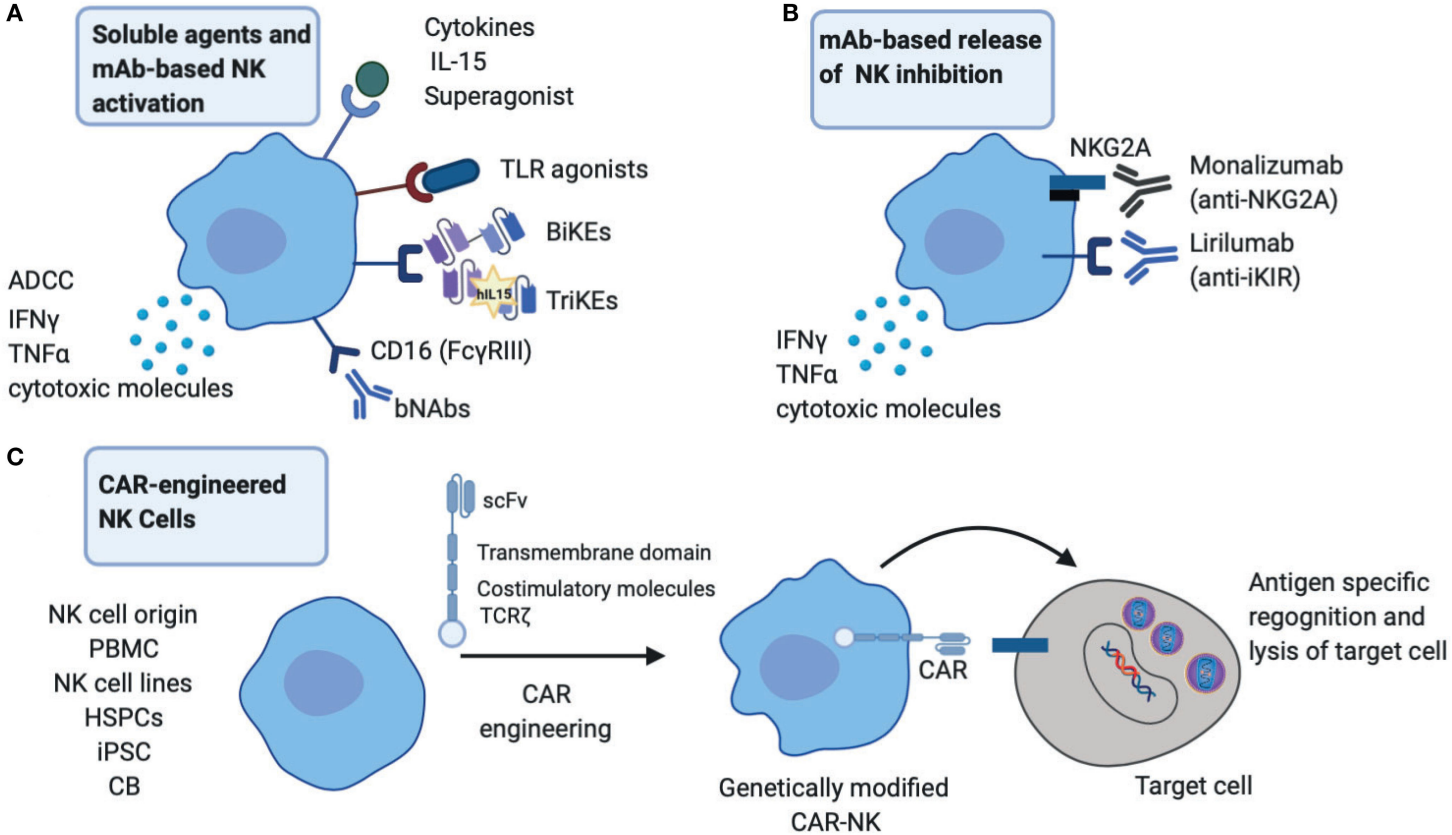
Therapeutic startegies to harness NK cells in HIV infection. **(A)** NK cell activation strategies through broadly neutralizing antibodies (bNAbs), engineered proteins, Bi-specific or Tri-specific Killer engagers (BiKEs or TriKEs), soluble mediators such as cytokines and TLR agonists to boost NK effector functions including cytotoxicity and cytokine production. **(B)** Release of NK inhibition via engagement of monoclonal antibodies (mAb) directly against inhibitory receptors NKG2A and inhibitory Killer-cell immunoglobulin-like receptor (iKIRs). **(C)** CAR-engineered NK cells to target HIV infected cells. PBMC, peripheral blood mononuclear cells; HSPCs, hematopoietic stem/progenitor cells; iPSC, induced pluripotent stem cells; CB, cord blood.

The success of NK cells in cancer immunotherapy is emerging as an exciting field in augmenting therapeutic approaches against chronic viral infections (Shimasaki et al., 2020). These are based on activating immunological mechanisms that would allow durable viral control by enhancing NK cell endogenous responses and/or generating new immune responses (Figure 1). An important consideration with such approaches continues to be a balance between promoting highly effective NK cell responses and abating any potential toxicity/bystander effects (Table 1).

**Table 1:** Selected trials and therapeutic approaches targeting natural killer (NK) cells in cancer and/or HIV1 immunotherapy and associated limitations of such approaches.

**Therapeutic approaches**	**Type**	**Mode of action**	**Limitations and challenges**	Trial registry identifier(s)^*^	**References**
Release of NK suppression	mAbs to NKG2A Monalizumab (previously IPH2201)	Blockade of NKG2A mediated inhibition of NK cells; synergizes with other checkpoint inhibitors or mAbs	Potential autoreactivity and off-target effects; optimal combination therapy	NCT02643550 NCT02921685 NCT02671435 NCT03822351 NCT03833440	Shimasaki et al., 2020
	mAbs to KIRs Lirilumab (IPH2102)	Blockade of inhibitory KIR mediated inhibition of NK cells	Potential autoreactivity and off-target effects; optimal combination therapy	NCT03532451 NCT01714739 NCT01687387 NCT01750580 NCT02252263 NCT02399917 NCT02481297	Ramsuran et al., 2018
Rev-up endogenous NK cell responses	BiKEs and TriKEs	Engage an activating receptor on NK cells (i.e., CD16), bridging it to a target cell; high efficacy; good safety profile	Complexity of design process; CD16 polymorphism; levels of CD16 expression on NK cells and CD16 cleavage	NCT01221571 NCT03192202 NCT03214666	Gleason et al., 2012; Rothe et al., 2015; Apps et al., 2016; Tay et al., 2016; Vallera et al., 2016; Sarhan et al., 2018a
	bNAbs	HIV neutralization; Fc mediated functions and ability to trigger NK cell-mediated ADCC	ADCC capacity of bNAbs; NK cell responsiveness; Fc receptor polymorphisms; possibility of generation of escape mutants	NCT02018510 NCT02825797	Halper-Stromberg and Nussenzweig, 2016; Li et al., 2017; Bar-On et al., 2018; Mendoza et al., 2018
Toll-Like receptor (TLR) agonists	TLR agonists	Enhance activation of components of adaptive and innate immunity, including NK cells	Potential off-target effects and toxicity	NCT02077868 NCT02200081 NCT02668770 NCT02858401 NCT03060447	Lu et al., 2016; Smith et al., 2018
Immunostimulatory cytokines +/- adoptive NK cell therapy	IL-2, IL12, IL-18, IL-15; IL-15 superagonists	*Ex vivo* cytokine NK cell expansion and activation; *In vivo* modulation and augmentation of NK cells responses	Systemic toxicity; optimal dosing required for expanded NK cells to prevent exhaustion; development of new compounds with improved pharmacokinetics	NCT01885897 NCT02191098 NCT04290546 NCT02890758 NCT03346499 NCT03899480	Romee et al., 2012; O'Sullivan et al., 2015; Huot et al., 2017
NK cell engineering	CAR-NK cell adoptive therapy; multiple cellular sources	Redirect NK cells against specific antigen to enhance lysis of target cells	Optimized CAR constructs to increase efficacy; remaining challenges to manufacturing and scaling up; potential toxicity	NCT02892695 NCT03056339 NCT03415100 NCT03941457	O'Sullivan et al., 2015; Romee et al., 2016; Oei et al., 2018; Angin et al., 2019

* *Trial Registry Identifier(s) in cancer/or HIV immunotherapy*.

## Release of NK Suppression

The use of monoclonal antibodies (mAbs) that target the interaction between MHC class I and NK cell inhibitory receptors represents one strategy that is currently used to enhance NK cell anti-tumor activity (van Hall et al., 2019). In the setting of HIV, HLA-E interaction with its ligand NKG2A expressed on NK cells could serve as an evasion mechanism to escape NK-mediated responses. In particular, elevated levels of HLA-A expression where shown to result in enhanced expression of HLA-E and increased NKG2A-mediated inhibition and subsequent impairment of HIV control (Ramsuran et al., 2018). Therapeutic blockade of NKG2A/HLA-E interaction could, therefore, improve HIV control alone or in combination with other approaches. In addition, blocking inhibitory signals mediated through inhibitory KIRs by Lirilumab (IPH2102) augment NK mediated elimination of autologous HLA-C expressing tumor cells (Romagne et al., 2009). Even though it is widely accepted that HIV downregulates HLA-A/B and most primary HIV isolates can mediate downmodulation of HLA-C (Apps et al., 2016) unleashing NK cells from inhibition, the extent of HLA-C downregulation and any residual binding to inhibitory KIRs could influence NK cell function (Korner et al., 2017). Hence the use of inhibitory KIR blockade could augment NK cell antiviral potency in curative strategies. In addition, NKG2A and inhibitory KIR blockade could have an effect on CD8 T cells expressing these receptors and work in a complementary fashion to increase cytotoxic T cell (CTL) activity. However, these approaches raise questions about excessive negative NK cell immunoregulation, potential autoreactivity and bystander killing of activated uninfected T cells.

## Rev-up Endogenous NK Cell Responses via BiKEs/TriKEs and bNAbs

One exciting approach of improving NK cell functionality is through the use of the immunomodulators termed Bispecific and Trispecific Killer cell Engagers (BiKEs and TriKEs) (Tay et al., 2016). These are small molecules consisting of a single chain variable fragment (scFv) from the heavy and light variable chains of an antibody connected to one (BiKE) or two (TriKE) variable portions of antibodies specific to an antigen expressed on the surface of target cells. One of the components targets CD16 expressed on NK cells to induce direct killing via ADCC (Gleason et al., 2012; Vallera et al., 2016). Their use circumvents previous challenges associated with NK cell-based therapies including: (i) lack of specific targeting by NK cells and (ii) limitations in NK cell activation, survival and expansion *in vivo* which is overcome by the more superior TriKEs with IL-15 supplementation (Vallera et al., 2016). These approaches are already gaining ground as potent pharmacological interventions in cancer (NCT01221571) (Rothe et al., 2015; Sarhan et al., 2018a). BiKEs consisting of CD16A binding antibody domains fused through a linker to an engineered one-domain soluble human CD4 with high affinity to HIV-envelope glycoproteins have also been reported to show potential in HIV treatment/eradication (Li et al., 2017).

Following the rise of single cell cloning techniques, next-generation anti-HIV-1 broadly neutralizing antibodies (bNAbs) with greater potency/breadth and the ability to suppress viral replication and potential for Fc-mediated clearance of virus-infected cells have now entered the clinical arena (Halper-Stromberg and Nussenzweig, 2016) with promising results (Bar-On et al., 2018; Mendoza et al., 2018). The capacity of bNAbs to potentially induce NK-mediated ADCC following passive immunization (Lu et al., 2016) is of immense interests in current HIV cure attempts, where the *in vivo* effect of bNAbs on NK cells in humans remains to be determined (upcoming RIO trial).

## Toll-Like Receptor (TLR) Agonists and NK Cell Activation

TLR engagement with respective agonists/ligands coordinate some of the innate effector responses mediated by NK cells during viral infections. As such, TLR agonists have been utilized for the enhancement of the immunotherapeutic potential of NK cells especially in cancer immunotherapy, where they are being tested as adjuvants in clinical trials (Smith et al., 2018). In the HIV cure field, studies in SHIV-infected non-human primates identified NK cell activation to be amongst the factors predictive of a delay in viral rebound in a vaccination regimen involving PGT121 bNAb infusion, a “kick” using TLR7 agonist (GS-9620), followed by antibody washout and treatment interruption (TI) (Borducchi et al., 2018). In this study, a proportion of PGT121 and GS-9620 treated animals remained undetectable for over 200 days following TI (Borducchi et al., 2018). These findings suggest a critical role of NK cells in strategies directed toward HIV eradication. Currently GS-9620 is being evaluated in two clinical trials, in HIV infected controllers (NCT03060447) and in individuals on suppressive ART (NCT02858401). These studies will provide valuable insights on safety, biological activity and impact on viral reservoirs.

## Immunostimulatory Cytokines

The role of IL-15 cytokine in maintaining NK cell maturation, proliferation, homeostasis and antiviral immunity is well-established (Cooper et al., 2002). Levels of IL-15 correlate with NK cell antiviral function and natural control of SIV replication in lymph node B cell follicles in African green monkeys, a likely viral reservoir (Huot et al., 2017). IL-15 is currently being tested in several clinical trials to improve NK cell function, persistence, and expansion (Guillerey et al., 2016). Remarkably, the IL-15 superagonist ALT-803 (also known as N-803) inhibited acute HIV infection through NK cell activation in humanized mice (Seay et al., 2015) and has been also shown to drive SIV-specific CD8 T cells to lymph nodes in macaques (Webb et al., 2018). ALT-803 is now being evaluated in a phase I clinical trial aiming to clear HIV-1 reservoirs (NCT02191098). The ability of IL-15 to enhance ADCC and augment NK cell mediated killing of HIV-infected target cells *ex vivo* (Garrido et al., 2018) could prove vital in the development of a functional cure for HIV. These findings, together with a recent report of IL-15 improving the efficacy of HIV-specific CD8 T cells from non-controllers (Angin et al., 2019), highlight the complementary effects of such an approach to simultaneously target and boost multiple arms of the immune response. IL-15 could synergize with other ongoing approaches that target NK cells. Along these lines, the safety and efficacy of IL-15 superagonists in combination with bNAbs to induce HIV control will be assessed in a future trial (NCT04340596).

## (CAR)-Engineered NK Cells and Adoptive NK Cell Transfer

Chimeric antigen receptor (CAR)-engineered NK cells offer great promise as a new immunotherapeutic tool in the HIV field. The recent success of CAR NK cells derived from cord blood transduced with a retroviral vector, expressing the genes encoding anti-CD19, IL-15, and a safety switch (inducible caspase 9), in patients with refractory or relapsed CD19 positive cancers, represents a remarkable achievement in the field (Liu et al., 2020). Importantly the administration of CAR-NK cells was not associated with the development of cytokine release syndrome and toxicity seen with the use of CAR-T cells (Liu et al., 2020). With limited success of CAR-T cells in clinical trials to suppress HIV replication (Deeks et al., 2002), exploitation of NK cells from different sources that can be modified by the use of CAR constructs has several benefits. These include scalability, *in vivo* persistence and more favorable side effect profile. CAR-NK cells could, therefore, represent an attractive alternative to T cell approaches. In a humanized mouse model, anti-CD19 CAR-modified hematopoietic stem/progenitor cells (HSPCs) could differentiate into effector NK cells mediating an innate antiviral response and protection against HIV (Zhen et al., 2015). No doubt in the years to come we will see more exciting developments in this area.

In addition to genetic engineering of NK cells, adoptive cell transfer therapies using *ex vivo* expanded autologous or allogeneic NK cells or NK cell lines, such as NK-92, to treat human cancers (Shimasaki et al., 2020) could be considered as a therapeutic tool for HIV. The safety and tolerability of adoptive transfer of haploidentical NK cells and IL-2 (NCT03346499) or IL-15 superagonist N-803 (NCT03899480) in HIV infection along with any measurable impact on viral reservoirs are currently being evaluated.

## The Case for Adaptive NK Cells in HIV Infection

The recognition that NK cell subsets can expand and form long-lasting pools of memory-like cells in response to viral infection resembling the enhanced responsiveness previously regarded as a unique feature of adaptive T and B cell responses, represents a major advance in the field of NK cell research. Several types of adaptive NK cells have been described in humans, including in response to cytokines, antibody-mediated stimulation, vaccination and CMV-derived peptides (O'Sullivan et al., 2015; Reeves et al., 2015; Nikzad et al., 2019).

The best characterized adaptive NK cell subset in humans, expressing high levels of the activating receptor NKG2C, the activating counterpart of NKG2A that also binds to HLA-E (with lower affinity than NKG2A) (Guma et al., 2006; Beziat et al., 2013; Peppa et al., 2018) is driven by CMV infection. Recently a highly specific recognition of certain CMV-encoded HLA-E presented peptides was elegantly demonstrated to promote adaptive NK cell expansions (Hammer et al., 2018; Rolle et al., 2018). In addition, a rare CMV derived UL40 peptide that is identical to the HLA-E-binding peptide in the HLA-G signal sequence, was reported to trigger optimal NK stimulation and to enhance NK cell ADCC responses (Rolle et al., 2018). The NKG2C^pos^ NK cell population largely overlaps with the FcεRIγ adaptor protein-deficient subset that expands in response to antibody-opsonized targets or immune complexes (Hwang et al., 2012; Zhang et al., 2013). These CMV-reactive adaptive NK cells are delineated by a distinct epigenetic signature, similar to memory CD8 T cells, and changes in receptor expression, key transcription factors and signaling adaptor proteins (Guma et al., 2004; Luetke-Eversloh et al., 2014; Schlums et al., 2015). They are functionally specialized with a more selective recognition repertoire (oligoclonal pattern of self HLA-C KIRs) and reduced ability to respond to bystander activation/engagement of multiple activating receptors (Hammer and Romagnani, 2017). Instead they favor strong Fc receptor-dependent effector functions, especially IFN-γ production (Schlums et al., 2015).

CMV co-infection is near universal in HIV infected cohorts. We and others have shown the strong influence of CMV co-infection/reactivation in shaping the NK cell repertoire during chronic HIV infection. This leads to an accelerated differentiation and adaptive reconfiguration of the NK cell pool and a bias toward CD16 mediated NK cell effector functions (Zhou et al., 2015; Peppa et al., 2018). In particular during HIV/CMV co-infection, UL40 or HLA-G derived peptides may stabilize the expression of HLA-E and fine tune NK cell activation and antibody driven adaptive responses (Cubero et al., 2020); this blurs the dichotomous effect of the genetic polymorphism at position −21 of HLA-B on NK cell function described in CMV seronegative donors (Horowitz et al., 2016).

These expanded adaptive NK cells in HIV infected patients display enhanced responses to overlapping HIV envelope peptides (Zhou et al., 2015). Several reports also suggest that CMV-associated (NKG2C+) adaptive NK cells influence the outcome of HIV infection and improve viral control (Thomas et al., 2012; Gondois-Rey et al., 2017; Ma et al., 2017). The presence of NK cells with mature/adaptive features (CD57+NKG2C+), during early HIV infection, is inversely correlated with HIV viral load and linked to lower viral set point, better early response to ART and better immunological outcome (Gondois-Rey et al., 2017; Ma et al., 2017). In contrast, NKG2C gene deletion is linked with higher HIV susceptibility and disease progression (Thomas et al., 2012).

In addition to the influence of CMV co-infection, a recent study demonstrated that the pro-inflammatory milieu in HIV infected patients drives the expansion of a defined NK subset with memory-like properties, characterized by CD94^+^CD56^hi^ and high expression of the transcription factor TCF7 (Wang et al., 2020). A combination of IL-12 and IL-15 was able to induce the generation of CD94^+^CD56^hi^ NK cells from CD94^−^CD56^+^ NK cells from HIV seronegative donors (Wang et al., 2020). These CD94^+^CD56^hi^ NK cells exhibited features in keeping with adaptive NK cells, including higher NKG2C expression, increased cytotoxicity and a more pronounced degranulation against HIV-infected CD4 T cells. The presence of NKG2C^+^TFC7^+^CD56^hi^ NK cells correlated with HIV-induced inflammation and altered homeostasis of the gut resident and circulating innate lymphoid cells (ILCs) (Wang et al., 2020). The effect of CMV reactivation in the gut and influence on local NK cell populations was not addressed in this study but important to delineate given the pronounced contribution of CMV reactivation to loss of intestinal epithelial integrity and chronic inflammation in HIV, despite suppressive ART (Maidji et al., 2017).

Antigen-specific NK cells have been reported in rhesus macaques infected with SIV/SHIV (Reeves et al., 2015) and emerging evidence also supports the existence of human HIV-specific memory NK cells developing in response to vaccination, homing to the liver and expressing a CXCR6+CD16^lo^CD69+T-bet^lo^Eomes^hi^ signature (Stegmann et al., 2016; Nikzad et al., 2019). Whether NK cell memory is restricted to resident populations (Paust et al., 2010; Nikzad et al., 2019) requires a deeper understanding of their relationship between blood and tissue and highlights the need for a closer examination of resident tissue populations in order to fully harness their therapeutic potential.

## The Advantage of Adaptive NK Cell-Based Immunotherapies

Adaptive NK cells make a significant proportion of the peripheral NK cell pool in HIV infection (Zhou et al., 2015; Peppa et al., 2018). In addition to their longevity, and *in vivo* persistence they are endowed with enhanced capacity for ADCC (Schlums et al., 2015). Thus, by exploiting the specificities of monoclonal antibodies (mAbs), adaptive NK cells can exhibit increased reactivity against different immunogenetic epitopes and these attributes could be exploited in combination with HIV bNAbs already in clinical trials (Mendoza et al., 2018). Adaptive NK cells also exhibit a mature profile, specifically lack expression of NKG2A and preferentially express self-HLA KIRs, supporting the notion that adaptive NKG2C+ NK cells will have a superior ability to recognize “missing-self” on HIV-infected targets that have downregulated HLA-C (Apps et al., 2016). In addition, they are more resilient displaying elevated resistance to myeloid-derived suppressor cells (MDSC) and Treg suppression (Sarhan et al., 2016, 2018b) and exhibit enhanced bioenergetic profile, including enhanced mitochondrial oxidative phosphorylation (OxPhos) relative to their conventional NK counterparts (Cichocki et al., 2018). Adaptive NK cells also display a distinct activation threshold. For instance, while T cell immunoreceptor with Ig and ITIM domains (TIGIT) (co-inhibitory receptor) has been associated with NK cell exhaustion, TIGIT expression in HIV infection marks a mature NK cell subset with adaptive traits and enhanced responses to virus-infected cells (Vendrame et al., 2020). Taken together adaptive NK cell subsets have unique immunological features that are highly desirable, especially at the sites of immune engagement.

A concern with utilizing bulk NK cells for immunotherapy is their potential for unwanted immunoregulatory functions, such as regulation of T cell responses (Peppa et al., 2013) and B cell responses via elimination of CD4 follicular helper (Tfh) cells (Bradley et al., 2018). Adaptive NK cells are poised toward less immunoregulation exhibiting decreased degranulation against activated uninfected T cells (Schlums et al., 2015) and limited ability to restrain virus-specific T cells (Duhan et al., 2019). Exploiting the properties of adaptive NK cell subpopulations could, therefore, have the additional benefit of sparing activated bystander uninfected T cells and enhancing virus-specific responses against HIV.

Importantly adaptive NK cells have *in vitro* expansion capability with predictable selectivity as an alternative or combination strategy for a functional cure. Utilizing HLA-E expressing transfectants has been a successful strategy for obtaining robust proliferation of functional adaptive NK cells, with profound skewing toward a single self KIR, and enhanced NKG2C effector potential against allogeneic acute lymphoblastic leukemia primary blasts (Liu et al., 2017). The exquisite specificity of adaptive NK cells to HLA-E presented peptides (Hammer et al., 2018; Rolle et al., 2018), which has been shown to influence proliferation and ADCC responses (Rolle et al., 2018), opens up the possibility for further refinement of these approaches by tailoring the peptide ligand on HLA-E. Various combinations of specific cytokines have also been demonstrated to induce expansions of cytokine-induced memory like NK cells with increased responsiveness (Romee et al., 2012). In particular, IL-15 stimulated bulk NK cells from HIV infected donors on suppressive ART, were able to recognize and clear autologous latently HIV infected CD4 T cells following exposure to the potent latency reversal histone deacetylase inhibitor (HDACi), Vorinostat (Garrido et al., 2018). Such an approach could therefore be tailored to direct/boost specific adaptive NK cell subpopulations for functional cure strategies but so far has remained unexplored.

Indeed, adoptive transfer of cytokine-induced adaptive NK cells are being tested in phase I clinical trials in AML patients (Romee et al., 2016). CAR-transduced mature/adaptive NK cells exhibited enhanced effector responses relative to other conventional NK counterparts (Oei et al., 2018) and CD19-CAR memory-like NK cells exhibited antigen-specificity, higher anti-tumor responses, and a greater expansion compared with classical CAR NK cells (Berrien-Elliott et al., 2019).

## Future Approaches and Concluding Remarks

The study of adaptive NK cell subpopulations and *ex vivo* expansion for clinical application represents an exciting new avenue for the development of novel therapeutic interventions in the field of HIV infection. These approaches can be combined with therapeutic antibodies improving their efficacy. In addition, the generation of memory NK cell represents a novel goal of new vaccination approaches incorporating targeted adjuvants or through enhancing presentation via HLA-E. Future innovative strategies for cure include manipulation of the metabolic machinery of immune cells and attempts to intrinsically rewire NK cells to improve their immunotherapeutic potential.

Despite the considerable amount of progress, additional work is required to fully unravel the unique properties of specialized and memory NK cells subsets, especially within key effector sites, along with their potential for functional exhaustion. This knowledge would be critical in order to leverage their distinct features and maximize their therapeutic use in chronic viral infections while offsetting any detrimental effects to adaptive immunity and the host.

## Author Contributions

AA, AO, and EM contributed to writing specific sections. DP edited the final version of the manuscript.

## Conflict of Interest

The authors declare that the research was conducted in the absence of any commercial or financial relationships that could be construed as a potential conflict of interest.
